# K13-propeller gene polymorphisms in *Plasmodium falciparum* parasite population in malaria affected countries: a systematic review of prevalence and risk factors

**DOI:** 10.1186/s12936-019-2701-6

**Published:** 2019-03-07

**Authors:** Moses Ocan, Dickens Akena, Sam Nsobya, Moses R. Kamya, Richard Senono, Alison Annet Kinengyere, Ekwaro Obuku

**Affiliations:** 1 Department of Pharmacology & Therapeutics, Makerere University, P.O. Box 7072, Kampala, Uganda; 2 Department of Psychiatry, Makerere University, P.O. Box 7072, Kampala, Uganda; 3 Department of Medical Microbiology, Makerere University, P.O. Box 7072, Kampala, Uganda; 4 Department of Medicine, Makerere University, P.O. Box 7072, Kampala, Uganda; 5 Infectious Disease Institute, Makerere University, P. O. Box 22418, Kampala, Uganda; 6 Albert Cook Library, Makerere University, P.O. Box 7072, Kampala, Uganda; 7 Clinical Epidemiology Unit, Department of Medicine, Makerere University, P.O. Box 7072, Kampala, Uganda; 8 Africa Centre for Systematic Reviews and Knowledge Translation, Makerere University College of Health Sciences, P.O. Box 7072, Kampala, Uganda; 9 Faculty of Epidemiology and Population Health, London School of Hygiene and Tropical Medicine, London, UK

**Keywords:** Artemisinin, K13-propeller gene polymorphism, Malaria, Africa, *Plasmodium falciparum*

## Abstract

**Background:**

Efficacy of artemisinin (ART) agents, a critical element of current malaria control efforts is threatened by emergence and spread of resistance. Mutations in *pfkelch13* gene associated with ART-resistance evolved in Southeast Asia (SEA). *k13* mutations whose role in ART-resistance remains unknown, have subsequently emerged independently across all malaria-affected regions. The aim of this systematic review was to determine the prevalence and identify risk factors of *Plasmodium falciparum k13* mutations in malaria-endemic countries.

**Methods:**

An electronic search of studies from 2014 to date was done in MEDLINE via PubMED, SCOPUS, EMBASE and LILACS/VHL databases. Mesh terms and Boolean operators (AND, OR) were used. Two librarians independently conducted this search (RS and AK). The articles were screened for inclusion using a priori criteria set following PRISMA-P and STREGA guidelines. Three independent reviewers (NL, BB, and OM) extracted the data. Data analysis was performed in Open Meta Analyst software. Random effects analysis (DL) was used and heterogeneity established using I^2^-statistic.

**Results:**

A total of 482 articles were retrieved from Pubmed = 302, Lilacs/Vhl = 50, Embase = 80, and Scopus = 37; Bibliography/other searches = 13, of which 374 did not meet the inclusion criteria. The aggregate prevalence of single nucleotide polymorphisms (SNPs) in *pfkelch13* gene was 27.6% (3694/14,827) (95% CI 22.9%, 32.3%). Sub-group analysis showed that aggregate prevalence of non-synonymous SNPs in *pfkelch13* gene was higher, 45.4% (95% CI 35.4%, 55.3%) in Southeast Asia as opposed to 7.6% (95% CI 5.6%, 9.5%) in the African region. A total of 165 independent *k13* mutations were identified across malaria-affected regions globally. A total of 16 non-validated *k13* mutations were associated with increased ART parasite clearance half-life (t_1/2_ > 5 h). The majority, 45.5% (75/165), of the mutations were reported in single *P. falciparum* parasite infections. Of the 165 *k13*-mutations, over half were reported as new alleles. Twenty (20) non-propeller mutations in the *pfkelch13* gene were identified.

**Conclusion:**

This review identified emergence of potential ART-resistance mediating *k13* mutations in the African region. Diversity of mutations in *pfkelch13* gene is highest in African region compared to SEA. Mutations outside the *pfkelch13* propeller region associated with increased ART parasite clearance half-life occur in malaria-affected regions.

## Background

The useful life of artemisinin (ART) agents in malaria treatment is at risk due to emergence of several resistance alleles in *pfkelch13* gene (*k13*) [1]. This is further worsened by the current lack of effective vaccine and alternative medicines to ART agents in malaria management. Decreased *Plasmodium falciparum* sensitivity to ART agents is rapidly evolving and expanding globally since being first reported in southeast Asia (SEA) [2, 3]. Widespread prevalence of parasite ART-resistance threatens current gains in malaria control and eradication efforts. This is especially the case as ART agents remain the only effective malaria treatment to date [4]. Diversity in *pfkelch13* gene polymorphisms hinders efforts to track and contain the threat of parasite ART-resistance especially in high malaria transmission regions. In addition, the role of a majority of reported African *k13* mutations in causing ART-resistance remains unknown [5].

An increase of 2 million malaria cases was observed in 2017 when 219 million cases were reported compared to 217 million in 2016 [4]. With malaria being one of the leading causes of morbidity and mortality especially in tropical countries, a further increase of an already high burden of malaria is a cause for concern. It is an indication of the need to rethink current malaria control efforts. With no widely effective vaccine, ART agents and their derivatives remain the only effective agents currently used in malaria control efforts. However, decreased *P. falciparum* parasite sensitivity that has emerged in SEA presents a threat to future efficacy of these agents.

Due to occurrence of multiple polymorphisms in *pfkelch13* gene, there is a need to regularly keep track of various emerging alleles in different malaria-affected regions globally. In this systematic review, the aim was to describe potential emergence and/or spread of ART-resistance alleles in *pfkelch13* gene across malaria-affected regions. The findings of this review are useful to malaria policy makers and research scientists as it provides a guide on potential *k13* mutations whose role in ART-resistance needs to be urgently established, especially in malaria-endemic regions outside SEA.

## Methods

### Protocol development

In 2017, a review protocol (#CRD 42018084624) was developed and registered in International Prospective Register of Systematic Reviews (PROSPERO: http://www.crd.york.ac.uk/prospero) and thereafter published it in peer review journal [6]. The recommendations of PRISMA-P [7] and STREGA guidelines [8] were observed during protocol development and review conduct.

### Review question

The review intended to answer the question: What is the aggregate prevalence of *pfkelch13* gene mutations in malaria-affected countries since change in malaria treatment policy to ART agents? [6].

### Search strategy

Two librarians (AK and RS) conducted independent search of MEDLINE via PubMED, SCOPUS, EMBASE, and LILACS/VHL databases. The search covered a period from 2014 to date. Database search was performed to identify studies that investigated prevalence of *k13* gene polymorphisms among *P. falciparum* parasites in malaria-affected countries. The following search terms were combined using Boolean operators “OR” for synonymous terms and “AND” across the elements of PECOS (Population, Exposure, Comparator, Outcomes and Study design). The terms used include, “*K13*-propeller gene polymorphisms”, “*K13*-polymorphisms”, “*K13*-gene polymorphisms”, “*K13*-propeller gene mutations”, “*K13*-mutation”, “Artemisinin resistance genes”, “Artemisinin resistance alleles” “Artemisinin resistance mutations”, “Artemisinin resistance polymorphisms”, “*Plasmodium falciparum*”, “Plasmodium parasite”, “Artemisinin”, “Artemether”, “Artesunate”, “Dihydroartemisinin”, “Artemisinin agents”, ACT, ART, “malaria-affected countries”.

Searching for articles in selected databases using the stated search terms was restricted to title or abstract. To maximize outcomes of the search, specific terms were not included such as those that focus on study design. The review found articles in French and Spanish, in which case Google translator (https://translate.google.com/) was used for translation to English prior to screening of the articles for inclusion.

### Additional searches

Screening reference lists of included studies was a key targeted article search approach. Further, a researcher in malaria (PR) was contacted for recent publications on *k13* gene polymorphisms in *P. falciparum* in different malaria-affected countries. There was no language restriction in article search and no search of grey literature.

### Data management

EndNote software version X7 (Thomson Reuters, 2015) was used in data management for the review articles. All identified titles of articles were imported into the software and duplicates removed. Article titles were then screened and grouped into different eligibility categories (included or excluded) following a priori criteria. Data extraction form was developed in Excel 2007, pre-tested on 5 articles and adjusted using findings of the pilot test. Data were extracted in triplicate and kept using the adjusted tool.

### Eligibility criteria for article selection

The review included articles from studies that investigated prevalence of *K13*-mutations among *P. falciparum* parasites in all malaria-affected regions/countries. Studies included were those that assessed for molecular markers of ART-resistance in *pfkelch13* gene. Studies that reported both molecular and phenotypic ART-resistance were included. Additionally, studies that assessed for *k13* mutations both before and after official introduction of ART combination therapy (ACT) in malaria treatment were included. Studies that analyzed for *k13* gene mutations using sequencing techniques were included. All studies that used cross-sectional and clinical trial designs in data collection were included.

### Exclusion criteria for ineligible articles

Studies that were excluded focused on *dhfr, dhps, pfAP2, PfATPase6/SERCA*, and *PvK12* resistance alleles, malaria treatment, malaria epidemiology and/or transmission, methods/drug targets, non-*Plasmodium* malaria parasites, pharmacogenetics, review/opinion papers, non-human participants, mathematical modelling of resistance, studies primarily done prior to official introduction of ART agents in malaria treatment, articles which reported only synonymous *k13* mutations, and citations whose full text articles could not be retrieved.

### Data extraction

Data extraction form was developed in Microsoft Excel 2007 (Microsoft Corp, Washington, USA) and extracted the following information from included articles: author, year of study, country/region, study design, sample collection method, laboratory where analysis was performed, allele calling algorithm, source of DNA, year when ACT was initiated, period/years covered by study, number of parasite DNA where genotyping was attempted, number of parasite DNA samples where genotyping was successful, *k13* mutations confirmed to cause delayed ART parasite clearance (validated SNPs in *k13* propeller mutations), non-validated SNPs in *k13* gene (*k13* mutations that have not been confirmed to cause delayed ART parasite clearance), non-propeller *k13* mutations, non-validated *k13* mutations associated with delayed ART parasite clearance, nucleotide changes in *k13* mutations, genotyping method used, duration of ART use, nature of ART use (monotherapy/combination) and source of *k13* mutation.

### Minimizing bias in article identification, selection and data extraction

A second librarian (RS) validated electronic search in PubMed by performing an independent and duplicate search. A second reviewer (EAO) screened all full text articles excluded by the first reviewer (MO). Any discrepancies among the reviewers were resolved by discussion and consensus. Two reviewers (NL and BB) performed duplicate and independent data extraction. Any disagreement between the reviewers was resolved by discussion and consensus. Any further disagreement between the two reviewers was referred to the third reviewer for a final decision (MO).

### Data synthesis

Extracted data were transferred to Open Meta Analyst software for analysis [9]. Both structured narrative and quantitative syntheses of extracted data were applied as appropriate. In the structured data synthesis descriptive summaries of outcomes of interest were generated. These included: non-synonymous SNPs in *pfkelch13* gene, duration of ART use prior to data collection, source of mutations (indigenous/introduced), nature of ART use (combination or monotherapy), allele calling algorithms, nucleotide changes in *k13* mutations, spread of *k13* mutaions, *pfkelch13* mutations not confirmed to cause delayed ART parasite clearance (non-validated) but associated with delayed *P. falciparum* parasite ART clearance, genotype success rate, frequency of *k13* mutations.

DerSimonian–Laird (DL) random effects analysis was used in establishing summary estimate of prevalence of non-synonymous SNPs in *pfkelch13* gene in malaria-affected regions using Open Meta Analyst software. Sub-group analysis was performed, region (SEA, Africa, India), and study design (cross-sectional, clinical trial designs). Heterogeneity in included articles was inferred from the summary estimates of I^2^-statistic. The I^2^ statistic was used to indicate percentage (%) heterogeneity that could be attributed to between-study variance. Interpretation: I^2^ = 25% (small heterogeneity), I^2^ = 50% (moderate heterogeneity), I^2^ = 75% (large heterogeneity) [10].

### Missing data and risk of bias assessment

The variables that were missing from included articles were recorded as not reported. Statistical tests were not used in handling missing data. However, authors whose articles had some missing variables were contacted but did not get any feedback.

The risk of publication bias was assessed using indirect assessment of rank correlation between effect size and sample size (Kendall’s tau) method. In this method correlation of the articles is interpreted from the analysis output in Open Meta (Analyst) software where 1, represent perfect correlation and 0 no correlation.

## Results

### Study identification and selection

**Fig. 1:**
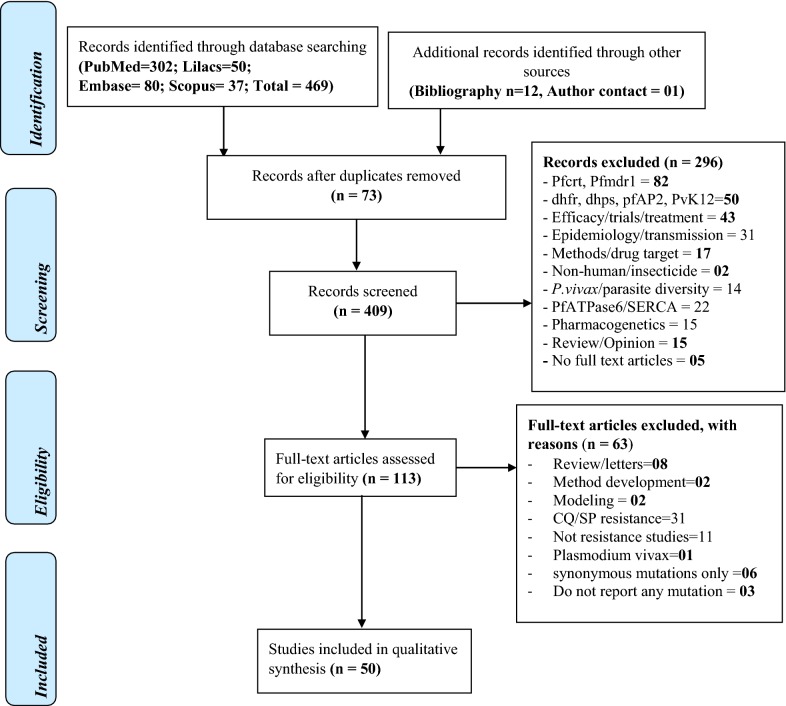
Prisma flow diagram showing article selection for the review

The search of Medline via PubMed, Embase, Lilacs/Vhl and Scopus databases yielded a total of 469 citations on *P. falciparum k13* gene single nucleotide polymorphisms (SNPs). Additionally, 12 citations were obtained from bibliography search and one article was obtained through contact with a malaria researcher (PR). After adjusting for duplicates, 409 citations remained. Of these, 296 articles were discarded after reviewing the titles and abstracts as they did not meet a priori inclusion criteria. Five citations were discarded as the full text articles could not be obtained. A total of 113 full text articles were screened in detail using pre-determined inclusion criteria. A total of 50 articles were included in the final full text review (Fig. 1).

### Characteristics of included studies

Twenty-five articles were from studies in SEA, 2 from India, 21 from Africa and one from studies both in Africa and China. Two studies, by Ashley et al. [11] and Menard et al. [12] were performed in multiple centers both in Africa and SEA. The reported study designs included: randomized control trials (3), cohort (2), non-randomized trials (2), and cross-sectional studies (43).

Over half, 52% (26/50) of included articles did not report the year of official initiation of ART use in malaria treatment in the region/country where data were collected. While in 24 studies that reported years of official introduction of ART use, the mean duration of time from official initiation of ACT use to data collection was 9.3 ± 7.2 years. In addition, countries where studies were conducted, ART agents had been in use in malaria treatment for on average 15.4 ± 7.7 years from official introduction to date (2018). The majority of studies on *P. falciparum k13* mutation were done after official introduction of ART agents in malaria treatment.

One study by Ariey et al. [1] used laboratory-adapted *P. falciparum* parasites from both SEA and sub-Saharan Africa while all other studies collected *P. falciparum* parasite isolates from symptomatic patients. ART agents were reportedly used as combination therapy in malaria treatment in all malaria-affected countries from where data were collected.

### Genotyping errors

Genotyping was conducted in total of 31,655 *P. falciparum* DNA isolates of which 27,667 (87.4%) were successfully genotyped (Table 1). The genotypes were assigned to successfully sequenced parasite DNA in small batches in all studies. Twenty-five studies collected *P. falciparum*-infected blood samples using filter papers (Whatman 903), EDTA vacutainer tubes (14 studies) while 11 studies did not report how samples were collected. The allele calling algorithms used included: Mega software *ver* 6.06, Bioedit *ver* 7.0.1, Sequencer software *ver* 5.4.5, Jalview software *ver* 5.1, Lasergene Genomic Suite, DnaSP software *ver* 5.50, Genome Assembly Program GAP4, Genescan, PROVEAN software tool, R software, Genotyper software and CEQ 2000 genetic Analysis System Software.

**Table 1:** Summary of the main findings from the reviewed articles

Article citation	Year	Design	Country	Valid K13-PM	Non-validated Non-synon. K13-PM (*amino acid changes*)	Sequence genotyping success rate (%)	Study period	Year when ART use was rolled out	Year of ART use *(from policy change to study)*	Prev. of parasite DNA with non-synon. K13-PM
*Southeast Asia*										
Ariey [1]	2014	CSS	Cambodia	R539T C580Y Y493HI543T	G449A N458Y T474I A481V T508N P527T G533S N537I P553L R561H V568G P574L S623C I543T P553L	94.2% (886/941)	2001–2012	NR	NR	33.9% (300/886)
Ashley [11]	2014	RCT	Asia, Africa	R539T C580Y Y493H I543T	P441L F446I G449A N458Y M476I A481V S522C N525D N537I G538V R561H V568G P574L Q613E A675V H719NN87K L143P K189N R255K G112E T149S R223K D281V E130G K189T E252Q K438N	82.1% (1019/1241)	2011–2013	NR	NR	32.9% (335/1019)
Bosman [52]	2014	CSS	Cambodia	C580Y Y493H	None	40.7% (11/27)	2013	NR	NR	63.6% (7/11)
Dwivedi [53]	2016	CSS	Cambodia	R539T C580Y Y493H I543T	V568G P553L	52.9% (282/533)	2010–2011	NR	NR	24.1% (68/282)
Imwong [44]	2015	CSS	East Thailand	C580Y R539T	Not reported	87.1% (88/101)	2014	NR	NR	93.2% (82/88)
Imwong [54]	2017	CSS	Greater Mekong sub region	C580Y R539T Y493H	F446I G449A N537I R561H A481V D584V C469F P441L P527H P553L P574L P667R R575K	100% (434/434)	2008–2015	NR	NR	93.2% (82/88)
Kheang [55]	2017	CSS	Cambodia	C580Y Y493H	Not reported	82.6% (76/92)	2014	2000	14 years	88.2% (67/76)
Leang [17]	2015	RCT	Cambodia	C580Y Y493H R539T	D584V P553L V568G	100% (419/419)	2011–2013	NR	NR	52.3% (219/419)
Mohon [56]	2014	CSS	Bangladesh	None	A578S	93.4% (253/271)	2009–2013	NR	NR	0.8% (2/253)
Thuy-Nhien [57]	2017	CSS	Vietnam	Y493H R539T C580Y	T474I P553L V568G P574L	100% (1060/1060)	2009–2016	2005	4 years	26.2% (278/1060)
Nyunt [15]	2015	CSS	Myanmar	C580Y R539T	M476I A481V N458Y R515T N537I G449A D452E K479I C469F R528T R575K V496F P441L	100% (91/91)	2013	NR	NR	31.9% (29/91)
Nyunt [28]	2017	CSS	Myanmar	C580Y	P574L P667T M476I	100% (100/100)	2013–2015	NR	NR	21% (21/100)
Nyunt [29]	2017	CH	Myanmar	C580Y	T474I M476V C469F N490T Y511H G533A G538V N537I P553L E556D R561H P574L F673I A675V	100% (550/550)	2009–2013	2002	7 years	33.6% (185/550)
Nyunt [58]	2017	CSS	Myanmar	C580Y	P574L	100% (4/4)	2015	NR	NR	50% (2/4)
Putaporntip [35]	2016	CSS	Thailand	C580Y R539T Y493H	P441L G449A N458Y A481V P527H E556D R561H P574L Y604H E605G N609S N632D	91.3% (272/298)	1991–2014	1995	BA/AF	67.3% (183/272)
Spring [16]	2015	CH	Cambodia	C580Y R539T	Not reported	100% (107/107)	2012–2014	NR	NR	96.3% (103/107)
Takala-Harrison [14]	2015	RCT	Southeast Asia	R539T C580Y Y493H I543T	P574L V568G P553L A481V G449A	55% (303/551)	2008–2011	NR	NR	40.6% (123/303)
Talundzic [37]	2015	CSS	Thailand	C580Y R539T	N458Y E556D P574L R575K S621F	100% (417/417)	2007	1995	12 years	12% (50/417)
Tun [30]	2015	CSS	Myanmar	C580Y	G449A N458I M476I F446I E252Q N537I R561H G538 V P574L A676D	39.5% (940/2378)	2013–2014	NR	NR	39.5% (371/940)
Wang [19]	2015	CSS	China–Myanmar	R539T	N11Y I352T I376V P443S C469Y L492S F495L K189T E252Q R255K P441L F446I N458Y C469Y P574L A676D H719N	100% (57/57)	2008–2013	NR	NR	66.7% (38/57)
Wang [38]	2015	CSS	China–Myanmar	C580Y	F446I N458Y C469Y P574L A676D H719N E252Q R255K F446I C469Y	94.2% (180/191)	2004–2012	1970	34 years	48.9% (88/180)
Win [21]	2016	CSS	Myanmar	None	P441L P443S G449A S485N Y511H A529T N554L P574L R561H R575K A675V A676D H719N I352T F446I E294G R254L	82.4% (206/250)	2013–2014	2005	8 years	36.9% (76/206)
Huang [36]	2015	CSS	South China	None	F446I F483S L492S F495L E556D	77.8% (329/423)	2007–2012	2009	BA/AF	47.7% (157/329)
Bonnington [13]	2017	RCT	Myanmar	C580Y	F446I R561H M476I I205T	91.4% (32/35)	2013	NR	NR	46.9% (15/32)
Menard [12]	2016	CSS	SEA, Africa	R539T C580Y Y493H I543T	F446I N458Y P574L R561H P553L A578S V589I S522C V534A F583L G665C N537D E605K	93.7% (13,157/14,037)	2012–2014	NR	NR	8.3% (1097/13,157)
*India*										
Mishra [59]	2015	CSS	India	None	G533A S549Y R561H A578S	98.7% (384/389)	2009–2013	2007	2 years	1% (4/384)
Mishra [60]	2016	CSS	India	None	F446I A578S K189T	100% (254/254)	2014	NR	NR	3.9% (10/254)
*African region*										
Balikagala [61]	2017	CSS	Uganda	None	A578S	97.9% (143/146)	2014	2006	8 years	1.4% (2/143)
Bayih [18]	2016	RCT	Ethiopia	None	A622I	84.5% (125/148)	2013–2014	2004	9 years	2.4% (3/125)
Boussaroque [22]	2016	CSS	Senegal	None	N554H Q613H V637I K123R N137S T149S K189T/N	89.3% (92/103)	2013–2014	2006	7 years	38% (35/92)
Conrad [20]	2014	CSS	Uganda	None	K189T K189N F334L I465T Y558H A578S A617T L619S V637D	76% (133/175)	2006–2012	NR	NR	10.5% (14/133)
Djaman [34]	2017	CSS	Ivory coast	None	E602D	100% (298/298)	2002–2013	2004	BA/AF	0.3% (1/298)
Dorkenoo [25]	2016	CSS	Togo	None	S522M A578S C532S S522C	95.6% (500/523)	2012–2013	2005	7 years	1.8% (9/500)
Escobar [62]	2015	CSS	Angola Mozambique	None	V494I	100% (200/200)	2003–2012	2004	BA/AF	1% (2/200)
Feng [33]	2015	CSS	Ghana–China	C580Y R539T	R575T D584V V692L	95.8% (113/118)	2013	NR	NR	8.8% (10/113)
Guerra [27]	2017	CSS	Equatorial Guinea	None	Y588C E556K A578S V637I D641G	93.5% (144/154)	2005–2013	2009	BA/AF	6.9% (10/144)
Gupta [63]	2018	CSS	Mozambique	None	P656I	100% (351/351)	2015	2004	11 years	2.8% (10/351)
Huang [64]	2015	CSS	Grande Comore Island	None	S477Y D584E D464H L488S A504T I526M A578S D584E	87% (180/207)	2006–2014	2004	2 years	11.1% (20/180)
Ikeda [39]	2018	CSS	Uganda	None	C469Y M472V A621S V666I A675V	100% (194/194)	2014–2016	NR	NR	2.6% (5/194)
Isozumi [23]	2015	CSS	Kenya	None	M442V N554S A569S A578S	57.5% (539/938)	2012–2013	2004	8 years	1.5% (8/539)
Kamau [5]	2015	CSS	SSA	None	A557S A578S L589I V566I A569T T630P S576L	100% (1212/1212)	2013–2014	NR	NR	1.2% (15/1212)
Li [65]	2016	CSS	Equatorial Guinea	None	A578S	90.7% (98/108)	2013–2014	NR	NR	2% (2/98)
Muwanguzi [66]	2016	CSS	Kenya	None	A578S	100% (69/69)	2009	NR	NR	1.4% (1/69)
Mvumbi [67]	2017	CSS	DRC	None	F495L V520A M476K N523T E509D	93.2% (261/280)	2014	2005	9 years	3.4% (9/261)
Ocan [68]	2016	CSS	Uganda	None	G533C S522A	60% (60/100)	2013–2014	2004	9 years	3.3% (2/60)
Ouattara [69]	2015	CSS	Mali	None	F446I A578S V581F D584N	98.6% (209/212)	1999–2011	NR	NR	12.4% (26/209)
Taylor [26]	2015	CSS	SSA	None	G449D W470X V520A C542Y G544R G545E S522C P553L A557S R561C A578S A617T G638R	100% (101/101)	2002–2011	NR	NR	49.5% (50/101)
Torrentino-Madamet [24]	2015	CSS	Mayotte, Comoros	None	N490H F495I N554H/K E596G	100% (29/29)	2013–2014	2004	9 years	17.2% (5/29)
Yang [31]	2017	CSS	China–Africa	R539T	M579I Q613E N664D A578S F662C N629S D464N D648Y E556K K610R A626T M476I V589I K658Q L663V P574L D648N	100% (483/483)	2012–2015	NR	NR	5% (24/483)
Tacoli [32]	2016	CSS	Rwanda	None	P574L A675V D648H V555A A626S	87.1% (222/255)	2010–2015	2005	5 years	2.3% (5/222)

NR, not reported; CSS, cross-sectional study; SSA, sub-Saharan Africa; RCT, randomized controlled trials; CH, cohort study; PM, propeller mutation; Non-synon., non-synonymous; Prev., prevalence; DNA, deoxyribonucleic acid

### Heterogeneity in the included studies

Heterogeneity was assessed in different sub-groups based on study design. The sub-groups included: cross-sectional studies (43), randomized control trials (RCT) (3: [11, 13, 14]), cohort (2: [15, 16]), and non-randomized clinical trials (2: [17, 18]). There was high heterogeneity across all sub-groups, cross-sectional studies (I^2^ = 99.38%, P < 0.001), RCTs (I^2^ = 74.4%, P = 0.02) and cohort studies (I^2^ = 99.81%, P < 0.001). Heterogeneity was still high when assessed across regions, SEA (I^2^ = 99.68%, P < 0.001), and Africa (I^2^ = 95.81%, P < 0.001).

### Codon positions where SNPs occur in *pfkelch13* gene

The majority, 86.7% (143/165) of observed non-synonymous SNPs occur within propeller region of *pfkelch13* gene. Of 84 non-synonymous SNPs in *K13*-gene in SEA, 66 (78.6%) are in propeller region (≥ 441) while 18 (21.4%) are out-side the propeller region (≤ 440) of *pfkelch13* gene. The non-synonymous SNPs in *k13* gene that occurred out-side propeller region in SEA included N87K (1 infected patient), G112E (1 infected patient), E130G (1 infected patient), L143P (1 infection), T149S (1 infection), K189T (31 infections), K189N (2 infections), R223K (1 infection), R255K (3 infections), D281V (1 infection), R254L (1 infection), I376V (1 infection) and E252Q (4 infections), K438N (1 infection), N11Y (1 infection), I352T (2 infections), E294G (1 infection), I205T (1 infection).

While of 76 non-synonymous SNPs in *k13* gene reported among sub-Saharan African parasites, 71 (93.4%) were in propeller region of *pfkelch13* gene. The *k13* mutations that occurred outside propeller region of *pfkelch13* gene included K123R (1 infected patient), N137S (1 infected patient), T149S (1 infected patient), K189N (1 infected patient), K189T (18 infected patients). The review observed that a total of 22 non-synonymous SNPs, 13.3% (22/165) in the *pfkelch13* gene occur outside the propeller region. In India, of the 07 *k13* mutations only one mutation K189T (1 infection) occurred outside the propeller region.

Double mutations in the same codon was reported at codon positions K189T/N [11, 19, 20]); N554L/H/S [21–24]); S522C/A [11]; [12, 25, 26]); P637I/D [20, 22, 27]); D648N/Y/H [21–24]); M476I/K/V [11, 13, 28–31] (Table 1). The nucleotide changes in codon positions with multiple mutations are, P637 → I, D (GTT → ATT or GTT → AAT), K189 → T, N (AAA → ACA or AAA → AAT), S522 → C, A (AGT → TGT or AGT → AGG), N554 → L, H or S (AAT → AGT, AAT → CAT or AAT → AGT) and D648 → N, Y or H (GAT → AAT, GAT → TAT or GAT → CAT).

### Overall prevalence of non-synonymous SNPs in *pfkelch13* gene

The overall prevalence of non-synonymous SNPs in *pfkelch13* gene (validated and non-validated) in reviewed articles from malaria-endemic countries is 27.6% (3694/14,827) (95% CI 22.9–32.3%). In SEA, the observed prevalence of validated *k13* mutations is 31.7% (1268/5328) (95% CI 19.3–44%). While the overall prevalence of non-synonymous SNPs (validated and non-validated) in SEA is 45.4% (4246/9352) (95% CI 35.4–55.3%). In African region the overall prevalence of non-synonymous SNPs (non-validated) in the *k13* gene is 7.6% (415/5450) (95% CI 5.6–9.5%). In India, overall prevalence of non-synonymous *k13* mutations is 2.2% (14/638).

### Epidemiology of non-synonymous SNPs in *pfkelch13* gene

Of 165 non-synonymous SNPs in *pfkelch13* gene reported in this review, 75 (45.5%) were found in single *P. falciparum* parasite infections. In SEA, 24 of 84 (28.6%) reported SNPs were found in single *P. falciparum* parasite infections. The validated *k13* mutation (confirmed to cause delayed ART parasite clearance) found in most malaria parasite infections (884) in SEA was C580Y. The non-validated k13 gene mutations occurring in a high number of *P. falciparum* infections in SEA include F446I (242), G449A (20), N458Y (22), 479 (30), 537 (31), P574L (100), A675V (28), G538V (36), and R561H (39). In Africa, 47 of 76 (61.8%) reported non-synonymous *k13* mutations were found in single *P. falciparum* parasite infections. The most common *k13* mutation, 40.8% (31/76) in Africa is A578S. The other non-synonymous SNPs in *pfkelch13* gene frequently reported among African parasite infections include 189T (18), D464H (09), D584V (05), V637I/D (05), N554L (04) and D648Y/H (03).

The *k13* mutation P574L that commonly occurs in SEA was reported in Rwanda [32] and in a study on immigrants from Africa to China [31]. The other mutations reported in low frequency in both Africa and SEA include A675V, P584L, R575K, 522C 189T, R561H, M476I, F446I, N554S, and A578S. Two studies by Feng et al. [33] and Yang et al. [31] in both Africa and China, reported a total of 21 different *k13* gene mutations of which 20 were found in only one parasite infection. Twenty of the 74 (27%) non-synonymous *k13* mutations in Africa were also reported in SEA. In India, of 5 reported non-synonymous *k13* mutations, 4 were found in only one *P. falciparum* parasite infection while a mutation, A578S was found in 2 *P. falciparum* infections in India. All the mutations found in India were also reported in other malaria-endemic regions. In China, the most commonly reported *k13* propeller gene mutations are F446I and P574L. The other SNPs reported in low frequency in China include H719N, M579I, F495L, L492S, C469F/Y, and N458Y (Table 1).

### Occurrence of *k13* gene mutations and ART use

In some studies in Africa, *k13* gene mutations were reported among *P. falciparum* parasites prior to official introduction of ACT in malaria treatment. These mutations include: E602D (Djaman et al. [34]: Ivory Coast); Y588C, E556K (Guerra et al. [27]: Equatorial Guinea). A study in Thailand by Putaporntip et al. [35] also reported existence of E605G and N609S mutations prior to introduction of ACT in malaria treatment. Of these mutations only one SNP at codon position E556K of *pfkelch13* gene persisted even after introduction of ART use in malaria treatment [27, 31]. A different allele at the same codon position 556 (E556D) was reported in SEA [29, 35–37].

Of 50 reviewed articles, 83% (34) indicated that the k13 mutations reported were indigenous to the region/country where the study was conducted. New SNPs in *pfkelch13* gene were reported each time in over half, 54% (27/50) of the studies. In SEA, ART agents have been in use in malaria treatment for close to five decades [38]. While in Africa, ACT has been used in malaria treatment for over a decade (~ 15 years).

### Non-validated, non-synonymous SNPs in *pfkelch13* gene associated with delayed ART parasite clearance

A total of 16 non-validated, non-synonymous *k13* mutations associated with delayed ART parasite clearance were reported by different studies, of which F446I (98 infections), P574L (61 infections), N458Y (22 infections), M476I (30 infections), A675V (28 infections), N537D (31 infections), and K189T (49 infections) were the most reported. A mutation at codon position A675V was reported in SEA, Uganda and Rwanda (East Africa) while Q613E and K189T were reported in different studies done in Senegal (West Africa) and SEA.

Non-synonymous SNPs in *pfkelch13* gene associated with delayed ART clearance among African *P. falciparum* parasites include: Q613H (Senegal [22], P574L (Rwanda [32]; China–Africa [31], A675V (Rwanda: [32]; Uganda: [39], P553L (Kenya: [26], and K189T (Senegal: [22]; Uganda: [20]) (Table 2).

**Table 2:** Non-synonymous K13-mutations associated with delayed ART parasite clearance

S/n	K13-mutation	No. of *P. falciparum* parasite infections	Region/country	References
1.	F446I	98	Malaysia, South China, India, Myanmar	[12, 13, 19, 30, 31, 36]
2.	P574L	61	Southeast Asia, Africa, Rwanda	[12, 14, 21, 30–32]
3.	N458Y	22	Asia,	[11, 12, 19]
4.	M476I	30	Myanmar	[28, 30]
5.	A675V	28	Rwanda, Uganda, Myanmar	[21, 32, 39]
6.	N537D	31	Myanmar	[29]
7.	R561H	12	Myanmar, India	[12, 29, 59]
8.	G538V	06	Asia	[11]
9.	I543T	05	Asia	[11]
10.	Q613E	01	Dakar, Senegal	[22]
11.	P553L	07	Southeast Asia	[12, 14, 17, 26, 29, 53, 54, 57]
12.	P667T	05	Myanmar	[28]
13.	C469F	11	Myanmar	[29]
14.	F673I	01	Myanmar	[29]
15.	K189T	49	Senegal, Uganda	[11, 19, 20, 22]
16.	E252Q	17	Southeast Asia	[11, 19, 30]

### Nucleotide changes of most reported non-validated *k13* gene SNPs

Single nucleotide polymorphism at codon position 574 (CCT → CTT) of the *k13* gene was reported by up to 5 different studies. The majority of frequently reported non-validated non-synonymous SNPs in *k13* gene occurred among African *P. falciparum* parasites (Table 3).

**Table 3:** Nucleotide changes for commonly reported non-validated K13-mutations

S/n	Codon position	Nucleotide change	Region/country	Article/study reporting the mutation
1.	A578S	GCT → TCT	China, Kenya, Grande Comore Island, Equatorial Guinea, Togo, Uganda, southeast Asia	[26, 31] [5, 23] [27, 64] [20, 25]
2.	S522C	AGT → AGG	Togo, Uganda, southeast Asia	[25, 68] [12]
3.	N554L	AAT → AGT AAT → CAT	Kenya, Senegal	[22, 23]
4.	P574L	CCT → CTT	Myanmar, Rwanda, Thailand	[28, 32] [5, 37] [12, 23]
5.	A569S	GCA → ACA	Sub-Saharan Africa	[5, 23]
6.	V637I	GTT → ATT	Equatorial Guinea, Senegal	[26, 27] [22]
7.	G449A	GGT → GCT	Sub-Saharan Africa Myanmar, Cambodia	[15, 26] [1]
8.	N537I	AAT → ATT	Cambodia, Myanmar	[1, 12, 15]
9.	D648H	GAT → AAT	China, Africa	[31, 32]
10.	A557S	GCA → TCA	Sub-Saharan Africa	[5, 26]
11.	A626S	GCA → TCA GCA → ACA	Rwanda China	[31, 32]
12.	M476V	ATG → ATA	China, Africa, Myanmar	[15, 29, 31]
13.	N458Y	AAT → TAT	Cambodia, Thailand	[1, 12, 37]
14.	G533C	GGT → TGT	Uganda, Cambodia	[1, 68]
15.	A617T	G → A	Sub-Saharan Africa	[20, 26]
16.	R575T	AGA → AAA	Thailand, Myanmar, Ghana, China	[15, 33, 37]
17.	V589I	GTC → ATC	China, Africa, SEA	[5, 12, 31]
18.	E556D	GAA → AAA	Equatorial Guinea, South China, Thailand, Africa	[27, 31, 36, 37]
19.	D584N	GAT → GAA GAT → GTT	Grande Comore Island Ghana, China	[64] [33]
20.	A675V	GCT → GTT	Rwanda, Uganda, Myanmar	[15, 32, 39]

### Non-synonymous SNPs in non-propeller region (< 440) of *pfkelch13* gene

The most frequently reported non-propeller *k13* mutation occurred at codon position K189T. The other common non-propeller *k13* mutations are located at codon positions 255, 252 and 352 of the *pfkelch13* gene. Non-propeller SNP at codon position K189T has been associated with delayed ART *P. falciparum* parasite clearance (*t*_1/2_ > 5 h*)* [11]. Mutation at codon position K189T was reported in both Africa and SEA (Table 4).

**Table 4:** Reported non-propeller K13-mutations in *Plasmodium falciparum* parasites

S/n	Study/article	Amino acid change/codon position (s)	Nucleotide change	Country/region
1.	[11]	N87K, T149S, E252Q, G112E, R255K, E130G, K189N, D281V, L143P, R223K	NR	Southeast Asia
2.	[13]	I250T	NR	Myanmar
3.	[22]	K123R, N137S, T149S, K189T	123 (AAA → AGA); 137 (AAT → AGT); 149 (ACT → TCT); 189T (AAA → ACA)	Senegal
4.	[20]	A189N, A189T, T334C	189T (A → C) 189N (A → T) 334 (T → C)	Uganda
5.	[54]	E252Q	NR	Greater Mekong Sub-region
6.	[60]	K189T	AAA → ACA	India
7.	[14]	R255K, K189N	189N (AAA → AAT)	Southeast Asia
8.	[30]	N371I, E252Q	NR	Myanmar
9.	[19]	E252Q, R255K	NR	China–Myanmar boarder
10.	[38]	N11Y, K189N, E252Q, R255K, I352T, I376V	NR	China–Myanmar boarder
11.	[21]	R254L, R255K, E294G, I352T	NR	Myanmar

NR, not reported

## Discussion

Malaria incidence rate reduced by 18% globally, from 76 to 63 cases per 1000 population at risk between 2010 and 2016 [4]. However, potential widespread emergence of *P. falciparum* parasite, mosquito resistance to ART agents and insecticides, respectively, is a threat to these gains in malaria control and elimination [4]. The review observed that overall prevalence of non-synonymous SNPs in propeller region of *pfkelch13* gene (both validated and non-validated) in malaria-affected regions is 27.6%. *k13* mutations confirmed to cause delayed ART parasite clearance (validated *k13* mutations) were observed in only SEA with aggregate prevalence of 45.4%. The observed prevalence of non-synonymous *k13* mutations varied in different regions with SEA, having the highest prevalence of *k13* mutations, while African region had higher diversity and low frequency of reported polymorphisms.

Since the first discovery of *k13* mutations as independent markers of ART-resistance in Western Cambodia [2] and later in Thai-Myanmar [40], there has been an overall upward trend in allele frequency globally [14]. In this review up to 165 *k13* mutations are reported across malaria-affected regions with 110 being reported in only one geographical location (SEA). Only 20% of the mutations reported in Africa were also detected in SEA. In addition, new mutations were reported each time in over 50% of the studies. This review highlights presence of diverse but low frequency occurrence of non-synonymous SNPs in *pfkelch13* gene across malaria-affected regions globally. In addition, the role of a majority of these alleles in parasite ART-resistance remains unknown.

Mutations R539T, C580Y (validated) and P574L (candidate) were reported in China-Africa studies. A study by Yang et al. [31] reported R539T and P574L among parasites from Angola and Equatorial Guinea, while Huang et al. [36] found C580Y and R539T among *P. falciparum* parasites isolated among immigrants to China from Ghana. These mutations could have been imported to China from Africa and is not clear whether they spread to Africa from SEA or emerged independently. A majority of the *k13* mutations arise independently as indicated by studies done in SEA [14, 41]. The current reported SEA alleles associated with ART-resistance among *P. falciparum* parasites observed in Africa and China could be due to independent emergence. Independent emergence of resistance alleles in *pfkelch13* gene potentially responsible for the observed variations in *k13* mutations across malaria-affected regions is influenced by several factors. These include widespread uncontrolled use of ART agents in addition to occurrence of anti-malarial medicines with sub-therapeutic amounts of active agents, especially in low and middle income countries. The extent to which different regions/countries are affected by these factors differs and could contribute to observed variations in *k13* mutations. Furthermore, existence of different parasite founder populations (genetic background) [42] in addition to variations in environmental conditions influences independent emergence of *k13* mutations.

Over half of the observed *k13* mutations were each reported in a single *P. falciparum* parasite infection. A study by Straimer et al. [43] showed that each single nucleotide change in *pfkelch13* gene is independently associated with an increase in parasite survival during ART exposure. Different *k13* propeller mutations mediate different levels of ART-resistance [16, 40]. Currently 6 ART-resistance alleles (N458Y, Y493H, R539T, I543T, R561H, C580Y) are confirmed (validated) as causes of ART parasite resistance [1]. Overtime a mutation at codon position C580Y has emerged as the most dominant in SEA and is associated with highest parasite clearance half-life [14]. The variation in extent to which these mutations affect ART parasite clearance half-life may be attributed to involvement of other loci in the genome [41, 42]. The numbers of *k13* mutations that are associated with increased clearance half-life of *P. falciparum* parasites by ART agents are rising over time. This has presented a challenge to policy makers and scientists who need to keep up with frequently validating different mutations. The review reports 16 *k13* mutations that are not validated (not confirmed) despite being associated with an increase in ART parasite clearance half-life. Some of the 16 potential ART-resistance alleles occur among African *P. falciparum* parasites (K189T, P553L, P574L, A675V, M476K, and Q613H). Despite the current association of some of these alleles with increased parasite clearance half-life, there is a need to validate all frequently reported SNPs in the *k13* gene among *P. falciparum* parasite populations in different regions, especially in Africa, which bears over 90% of malaria burden. A recent study by Mukherjee et al. showed that parasites carrying *k13* mutation D584V had increased in vitro RSA_0–3 h_ survival. The mutation D584 V was reported in SEA [17, 44] and among African migrants to China [36].

In the current review, 20 of 165 observed *k13* mutations occur outside propeller region of *pfkelch13* gene. Two of these mutations (A189T and E252Q) have been associated with increased ART parasite clearance half-life [42]. A mutation in codon position K189T was reported in both SEA and Africa. Molecular screening for ART-resistance currently is based on sequencing propeller region of *pfkelch13* gene (1, 725,980–1726, 940 bp; amino acid positions 419–707) [1]. However, this review shows that studies have recently established existence of non-synonymous mutations outside the propeller region of *pfkelch13* gene that are associated with increased ART parasite clearance half-life. In Cambodia, a mutation E252Q was reported by Wang et al. [19], Tun et al. [30], Ashley et al. [11], while D281V and K189T were in a study by Ashley et al. [11]. In Africa, mutation K189T was reported by Boussaroque et al. [22] (Senegal) and Conrad et al. [20] (Uganda). These mutations may provide valuable clues for the existence of gene polymorphisms potentially associated with ART-resistance outside the propeller region of *pfkelch13* gene [42].

On the Thai-Myanmar border, ACT was first introduced in malaria treatment in 1994 with resistance suspected just over one decade later, in 2008, and subsequently confirmed in a study by Ariey et al. [1]. The review observed non-synonymous *k13* mutations among *P. falciparum* parasites that occurred prior to official introduction of ART agents in malaria treatment. These included: E602D, I588C, E252Q, and E556K and were all reported in African *P. falciparum* parasites. Only one of these mutations, E556K, persisted in parasite population even after official introduction of ART agents in malaria treatment. A study by Hermission [45] indicated that ART-resistance alleles in *k13* gene became established within a year of official ART deployment in malaria treatment in SEA. In malaria-affected countries outside SEA however, after over a decade of ART use in malaria treatment, there is still no confirmed *k13* resistant alleles among parasite population. In Africa, which bears over 90% of malaria burden, this has been welcome news. However, current resistance surveillance reports have indicated potential emergence of parasites with increased ART clearance half-life in the region.

Although ART agents with effective partner drugs still cure patients harbouring slow ART clearing parasites [14, 46], resistance development to ART component of the combination will expose more parasites to partner drug alone, increasing likelihood of ACT failure. The current failure by dihydroartemisinin-piperaquine combination to cure patients in Western Cambodia is a demonstration of this phenomenon [14]. The review observed that ART is used as combination agents in malaria treatment across malaria-affected countries. The use of combination ART agents (ACT) in malaria treatment potentially reduces resistance development [47]. However, eradication of ART-resistant malaria parasites necessitates complete elimination of all malaria since, as malaria incidence reduces, the few infections which remain to be cleared are almost all resistant [47]. Using long-lasting insecticide-treated mosquito nets alongside ACT could go a long way in eradicating malaria disease due to the synergistic effect on malaria incidence [47].

Use of ART agents in malaria treatment influences development of parasite resistance. The review observed emergence of multiple *k13* mutations across malaria-affected countries following introduction of ART agents. A study by Amaratunga et al. [48] showed that ART agents select for resistance among *P. falciparum* parasites. In addition, it is probable that use of partner drugs such as mefloquine [49], piperaquine [14], and lumefantrine [50] to which the parasites are resistant may be influencing ART-resistance development. The magnitude of ACT selective pressure on development of parasite ART-resistance is thus likely to be greater, especially in infections with concomitant resistance to partner drugs [47]. The current malaria combination therapy may benefit from having more than two active pharmaceutical ingredients. The use of combination therapy having more than two active ingredients has proved effective in restraining HIV resistance development [51]. However, treatment of infectious diseases in most low and middle income countries is faced with the challenge of limited therapeutic options affecting potential use of more than two active ingredients in malaria combination therapy.

The review had some limitations, few studies were retrieved and reviewed from India and none from Central Asia and South America. This was mainly due to poorly designed studies, which were excluded. However, the application of extensive literature search and a priori selection criteria reduces the potential effect of this limitation on the overall review outcome. Most of the articles poorly reported their findings with some omitting a few review variables. To reduce the effect of missing variables, outcomes were recalculated for the review variables using data reported in the articles. In addition, corresponding authors of the articles were contacted for missing information, although no response was received.

## Conclusion

The number of potential ART resistance alleles in the *pfkelch13* gene is rising across malaria-affected regions. The *k13* mutations with potential role in ART-resistance have been reported to occur outside the propeller region of *pfkelch13* gene. Among African *P. falciparum* parasites although not validated, *k13* mutations associated with delayed parasite clearance have been reported. Overall prevalence of non-synonymous SNPs in the *k13* gene is higher in SEA with higher diversity observed among African parasites. *k13* mutations occurring in more than one geographical region affected by malaria is increasing, however, polymorphisms associated with ART resistance have not yet been observed outside SEA.

## Abbreviations

PRISMA: preferred reporting items for systematic reviews and meta-analysis
STROBE: strengthening reporting of observational studies in epidemiology
PROSPERO: International Prospective Register of Systematic Reviews
STREGA: Strengthening the Reporting of Genetic Association Studies
